# Middle-Range Theory for Risk-Prone Health Behavior in College Students

**DOI:** 10.1590/0034-7167-2025-0664

**Published:** 2026-09-21

**Authors:** Pedro Lucas Ferreira Mota, Lucas Dias Soares Machado, Adriana de Moraes Bezerra, Maria Sinara Farias, Viviane Martins da Silva, Nayara Santana Brito

**Affiliations:** I Universidade Federal do Ceará. Fortaleza, Ceará, Brazil; II Universidade Regional do Cariri. Iguatu, Ceará, Brazil; III Universidade Regional do Cariri. Crato, Ceará, Brazil; IV Universidade Estadual Vale do Acaraú. Sobral, Ceará, Brazil

**Keywords:** Nursing Theory, Health Risk Behaviors, Students, Universities, Nursing., Teoría de Enfermería, Conductas de Riesgo para la Salud, Estudiantes, Universidades, Enfermería.

## Abstract

**Objectives:**

to describe the development of a Middle-Range Theory for Risk-Prone Health Behavior in College Students.

**Methods:**

this theoretical study is geared towards developing a theory by combining inductive techniques through conceptual analysis and focus groups, and by deriving concepts from Dorothy Johnson’s Behavioral Systems Theory.

**Results:**

the theory articulates eight primary concepts, ten secondary concepts, ten assumptions, and ten propositions that, together, seek to describe and explain the college context, its constitutive dimensions, and the manifestation of health conduct, health behaviors, risk-prone health behaviors, and risk behaviors.

**Final Considerations:**

the theory provides a theoretical and practical basis that overcomes the reductionist view of risk-prone health behaviors in college students, promoting an integrated analysis of the individual, their determinants and behavioral stages, and guiding nursing practice.

## RESUMEN

**Objetivos:**

describir el desarrollo de una Teoría de Rango Medio sobre el Comportamiento de Riesgo en materia de salud en estudiantes universitarios.

**Métodos:**

estudio teórico orientado a desarrollar una teoría mediante la combinación de técnicas inductivas a través del análisis conceptual y los grupos focales, y mediante la derivación de conceptos de la Teoría de los Sistemas de Comportamiento de Dorothy Johnson.

**Resultados:**

la teoría articula ocho conceptos primarios, diez conceptos secundarios, diez supuestos y diez proposiciones que, en conjunto, buscan describir y explicar el contexto universitario, sus dimensiones constitutivas y la manifestación de la conducta en materia de salud, los comportamientos relacionados con la salud, los comportamientos de riesgo para la salud y los comportamientos de riesgo.

**Consideraciones Finales:**

esta teoría proporciona una base teórica y práctica que supera la visión reduccionista de las conductas de riesgo para la salud en los estudiantes universitarios, promoviendo un análisis integrado del individuo, sus determinantes y etapas conductuales, y guiando la práctica de enfermería.

## INTRODUCTION

The process of adapting to college life is complex and requires students to make several adjustments to their routine, as well as adopting and maintaining healthy habits and overcoming obstacles, especially in the area of health^(1)^. The manifestation of health risk behaviors is recognized as a product of this process, characterized by a predisposition to morbidities resulting from common practices in this population, such as sedentary lifestyle^(2)^, inadequate eating habits^(3)^, impaired sleep patterns^(4)^, risky sexual behaviors, substance abuse, and dangerous behaviors in traffic^(5)^. Despite the extensive literature on these behaviors, there is still a scarcity of theoretical models that systematically explain the mechanisms that predispose college students to risky practices, especially from a nursing perspective.

Nurses’ understanding, recognition, and action in relation to risky health behaviors among college students demonstrate sensitivity to the needs of this group and the establishment of proactive strategies that promote well-being according to their real demands. Therefore, the implementation of assertive and transformative actions capable of supporting care practices consistent with the unique characteristics of this population becomes essential^(6)^.

To effectively implement the actions, the Nursing Process is proposed as a methodological, organizational, and dynamic tool, structured in five interrelated, interdependent, recurring, and cyclical stages: assessment; nursing diagnosis; planning; implementation; and nursing progress notes^(7)^. In turn, it is relevant that this process be anchored in nursing theories that describe, explain, predict, or prescribe the conditions and connections of a phenomenon, such as risk-prone health behaviors in college students, thus qualifying clinical practice and strengthening disciplinary identity^(8)^.

Although significant advances have been observed in the theoretical field of nursing, the application of these frameworks to the care of college students remains incipient. This gap highlights the need to construct a specific model that supports nurses’ practice and broadens the understanding of the health-disease process in this context.

Middle-Range Theories (MRTs) stand out in this scenario due to their practical applicability and potential to bridge the gap between theory and practice. Conceptually, they encompass a set of concepts with a lower level of abstraction and operational definitions, constituting concrete propositions that are amenable to empirical verification^(9,10)^. This characteristic reinforces its epistemological value for the advancement of knowledge in nursing.

To date, there are no records of MRTs specifically focused on risk-prone health behaviors in college students that integrate concepts and definitions aimed at describing and explaining this phenomenon and its relationships with the multiple stages of the health-disease process. This gap underlies the proposal of this study, highlighting its unprecedented and innovative nature.

It is therefore postulated that the development of a MRT focused on risk-prone health behaviors in college students will allow the construction of a theoretical framework capable of describing and explaining the units that compose the phenomenon, as well as their interrelationships with other categories of human behavior. Furthermore, this theory will have the potential to underpin nursing actions and intervene in the factors that predict and cause illness.

The proposed approach also aligns with the Sustainable Development Goals (SDGs), especially SDG 3 (Good Health and Well-being), which aims to ensure healthy lives and promote well-being for all people at all ages. More specifically, it relates to SDG 3.5, which proposes strengthening the prevention and treatment of substance abuse, and SDG 3.8, which seeks to guarantee universal health coverage, as well as access to quality services and medicines at affordable prices. These connections reinforce the social and political relevance of the proposal when addressing risk-prone health behaviors and their implications for healthcare.

## OBJECTIVES

To describe the development of a MRT for risk-prone health behavior in college students.

## METHODS

### Ethical aspects

The research was approved by the *Universidade Regional do Cariri* Research Ethics Committee according to the Certificate of Presentation for Ethical Consideration 78484824.3.0000.5055.

### Theoretical framework

This study adopts Johnson’s Behavioral System (JBS) as a theoretical framework, since this system articulates concepts such as subsystems, structural components, and functional requirements, which allow us to explain the relationships of individuals, as a behavioral system, with themselves, among their subsystems, and with the environment. This theory highlights nursing as a force promoting balance, capable of intervening in behavioral dysfunctions and favoring their harmony^(11)^, configuring itself as a pertinent foundation for the phenomenon under study. Her writings are part of the systems era, marked by the search for understanding the whole, and aimed to reaffirm nursing as an autonomous profession distinct from other areas of health.

### Study design

This is a theoretical study, aimed at developing a MRT, based on a combination of induction and derivation techniques.

### Methodological procedures

The theorizing process employed inductive techniques through conceptual analysis and focus groups, as well as the derivation of a grand theory^(10)^.

Conceptual analysis sought concepts in the scientific literature regarding risk-prone health behavior in college students through an integrative review, resulting in the delimitation of the units that comprise the concept^(12)^. In this regard, antecedents, attributes, consequences, conceptual definitions, cases, and empirical indicators were incorporated into theorization.

In order to understand the subjectivity of the phenomenon’s manifestation, focus groups were conducted with students from a public college in the state of Ceará and professionals providing care to college students from a public institution in Paraíba. The use of participants from different institutions was due to the authors’ connection during study development, access to college students at one institution, and the existence of student support centers at the other. Thus, the participation of 30 college students in three groups was recorded, with an average time of 46 minutes, and 16 professionals representing the student care centers of the institution’s campuses, in a single group, with an approximate duration of two hours. Data saturation and the thematic area marked the end of data collection with college students, while representation from 11 campuses of the institution in Paraíba was achieved at the time with professionals (52.3%).

The text *corpora* were analyzed using the *Interface de R pour les Analyses Multidimensionnelles de Textes et de Questionnaires* according to Descending Hierarchical Classification. In this way, conceptions of the phenomenon were considered in the theorization to refine the definitions, as well as the relationships between behaviors.

Thus, the material derived from the analysis of concepts and focus groups supported the formulation of statements, which serve to underpin the conception of the theory. These statements can be relational, establishing connections between two or more concepts of a theory and revealing correlations, or non-relational, constituting theoretical or operational definitions of a concept^(10)^.

Finally, comparisons were made between the primary framework and the JBS, complementing, contrasting, or reinforcing them, based on theoretical and empirical evidence. This process is characterized as derivation, a creative strategy based on analogy and the transposition of ideas from one theoretical field to explain and/or predict in a new field^(10)^.

In the final stage, theoretical modeling was carried out, resulting in a pictorial scheme of the MRT. As a product, the theory, its purposes, concepts, and non-relational statements were presented, along with relational statements between the concepts, assumptions, propositions, and the pictorial model, enabling an understanding of the theory’s context and its potential applications.

## RESULTS

### Middle-Range Theory for Risk-Prone Health Behavior in College Students

The Middle-Range Theory for Risk-Prone Health Behavior in College students (*Teoria de Médio Alcance para o Comportamento de Saúde Propenso a Riscos em Universitários* -TECompSPRU) is a scientific theory of intermediate level of abstraction (MRT), descriptive and explanatory in nature, developed from an *etic* (external) perspective, which incorporated evidence from studies with college students conducted in different national and international contexts, and an *emic* (internal) perspective, which involved the subjectivities of professionals working in the care of college students and representatives of the population of interest itself. These perspectives supported the purpose of contributing to the formulation, consolidation, and expansion of the body of knowledge in contemporary nursing, focusing on the care of college students, preceding theoretical development.

### Theory purposes

TECompSPRU seeks to describe and explain the relationships between college students and the manifestation of behaviors, especially risk-prone health behaviors, aiming to provide a framework for understanding the construct and nursing care.

### Non-relational concepts and statements

The identification of concepts precedes the formulation of definitions. Thus, based on evidence built throughout the study, primary concepts (college; subsystems; stressors; health conduct; health behavior; risk-prone health behavior; risk behavior; and nursing care) and secondary concepts (personal and individual development subsystem; relational subsystem; formative subsystem; psychological subsystem; behavioral subsystem; socioeconomic subsystem; healthcare subsystem; structural factors; maintenance factors; and transition) were delimited. Conceptual definitions were formulated, especially adapting the central units of the concepts behavioral system (represented in this theory by the college concept), subsystems, and stressors, addressed by Dorothy Johnson in her theory.

Therefore, the conceptual definitions that comprise TECompSPRU are listed below.

Aligned with each other, the system refers to a whole that is stabilized by the adequacy of its parts, and behavior constitutes voluntary or involuntary responses influenced by the environment, whether it be the personal being or the environment of interaction. In contrast, college students refer to a behavioral system that, through the interaction of its parts, produces voluntary or involuntary, repetitive and/or organized responses capable of establishing relationships with the community and the environment.

**Figure 1:**
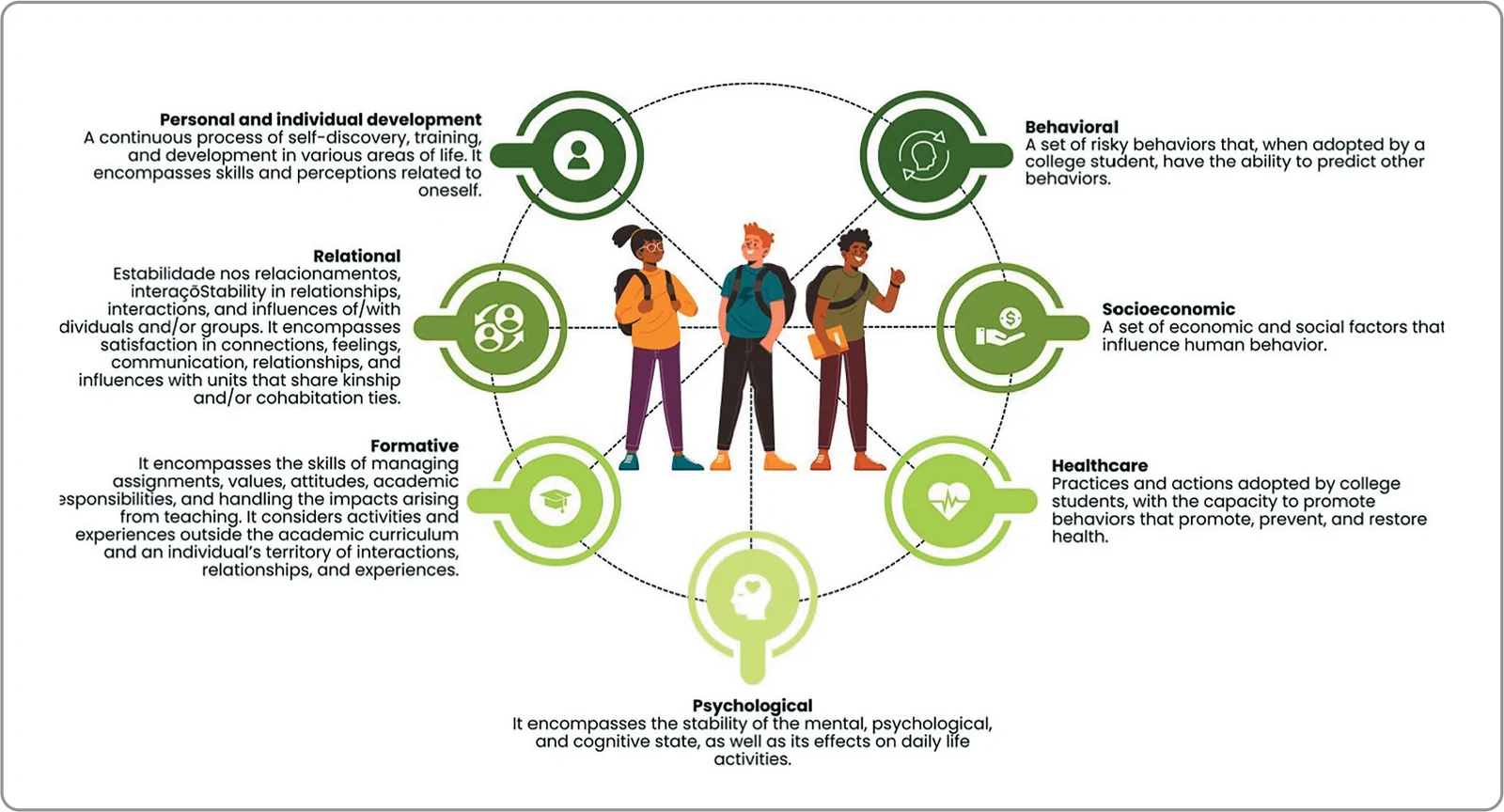
Definitions of the subsystems that comprise the Middle-Range Theory for Risk-Prone Health Behavior in College students, Iguatu, Ceará, Brazil, 2026

The subsystems are interconnected units, grounded in the individuality and events of higher education, responsible for the harmony of the whole. These are punctuated and defined as shown in Figure 1.

The functioning of the subsystems is mediated by structural factors, involving criticality and assessment between: situation (stimulus/stressor); judgment (situation assessment and goal establishment); and behavior (operationalization). In addition, maintenance factors provide support for maintaining behavioral effectiveness, comprising three elements: (1) health knowledge (promotes health behaviors and health conduct); (2) social resources (enable the adoption of health behaviors); and (3) support (involves environmental/institutional and relational encouragement).

In turn, stressors are unfavorable stimuli originating from the individual or the college environment, which are associated with risk-prone health behavior and/or risky behavior, given the multi-causality and inseparability of their constituents. In other words, the units that make up the three axes will constantly trigger new behaviors.

Regarding the possible stages that a college student can go through based on their attitudes, the following stand out: health conduct, which is the state or action resulting from personal, family and/or cultural upbringing that, consciously or not, acts beneficially in health processes; and health behavior, which refers to a set of conscious practices that maintain or promote healthcare; health behaviors prone to risks are defined as tendencies toward adopting habits that are harmful to health and well-being, and which are voluntary, negative, unfavorable, and/or destructive in nature; and risk behaviors are characterized as recurrent and well-defined behavioral patterns that promote illness.

Transition is the ability of a college student to move between stages of health conduct, health behavior, risk-prone health behavior, and risk behavior. This journey can be mediated by: (1) quantity of stimuli - single (involves one stimulus) or multiple (involves more than one stimulus); and (2) temporality - punctual (occurs in a short period, such as hours and days), limited (well-defined period within the vital programming and organization of the system), and constant (longer duration, sometimes recurring and with poorly defined deadlines).

Therefore, nursing care involves ordered and systematic actions, based on clinical reasoning and decision-making, aimed at prevention (minimizing the stimuli of illness), promotion (improving quality of life and autonomy to cope with predictors that favor illness), and recovery (care and management in the face of illness) of college students’ health. These actions should be mediated by the organizational and management tools of nursing practice, and implemented primarily in maintaining or restoring college students’ well-being.

Linked to these concepts, a connection was established with Johnson’s metaparadigm of nursing (person, health, environment, and nursing), given its essential role in shaping the boundaries of the discipline’s knowledge in comparison with other health-related fields (Chart 1).

**Chart 1:** Representations of Johnson’s nursing metaparadigm in the Middle-Range Theory of Risk-Prone Health Behavior in College students, Iguatu, Ceará, Brazil, 2026

Nursing metaparadigm	Definition in the Middle-Range Theory for Risk-Prone Health Behavior in College students
Person	College students are a subjective, adaptable, unique, and relational behavioral system, whose interaction with the college predisposes them to the occurrence of risky health behaviors.
Health	It integrates the individual state and the pursuit of well-being and harmony of college students in its multiple aspects, requiring that care be articulated with prevention and health promotion in the face of factors that predict the phenomenon or recovery of health in situations of illness.
Environment	It considers the individuality of college students for the manifestation of human responses, as well as the moderating or confounding stimuli of higher education that contribute to the occurrence of the event.
Nursing	It is the science of holistic care, based on theoretical and practical foundations and distinct from medicine. It comprehensively addresses college students in their physical, emotional, and social dimensions. Its scope includes welcoming, mapping predictors, clinical reasoning, decision-making, and the application of interventions aligned with the reduction/control of factors that potentiate the event, as well as the recovery from associated illness conditions.

### Relational statements between the theory concepts

**Figure 2:**
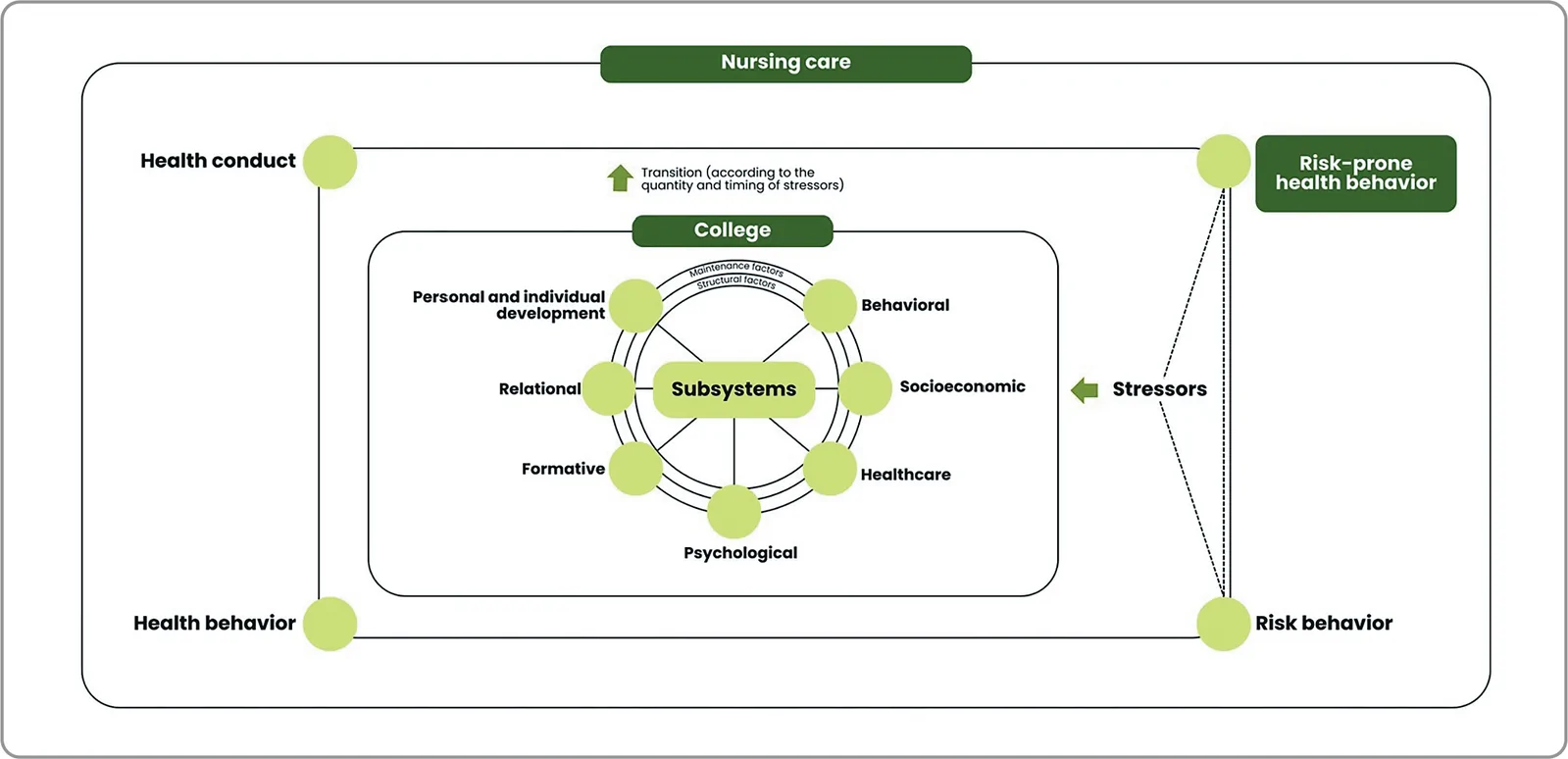
Relational statements between the concepts that comprise the Middle-Range Theory for Risk-Prone Health Behavior in College students, Iguatu, Ceará, Brazil, 2026

Conceptual relationships were, in summary, presented in Figure 2.

College students are a behavioral system that, through harmony (health) or disharmony (illness/aggravation) between its subsystems, whether through stimuli from college students themselves or from the college environment, adopts attitudes aligned with health behaviors, healthy behavior, risk-prone health behavior, and/or risky behavior.

Risk-prone health behavior is considered the central focus of this proposition, given that minimizing its occurrence leads college students (person) to behaviors that promote health, i.e., it reduces risky behavior and, consequently, illness. Conversely, the exacerbation of these behaviors promotes illness.

In this way, nurses, as a holistic and competent professional in the implementation of nursing care, must provide college students with strategies that eliminate stressors, promote health (health maintenance), and, in cases of recurrence of risk-prone behaviors, act in a way that provides support for health recovery. It is relevant that nursing care be aligned with the ethical responsibilities of the profession and based on scientific evidence, encompassing everything from welcoming and identifying stressors to prescribing and verifying the effectiveness of a care plan. Thus, nursing aims to understand the factors that destabilize college students and to promote the process of health and well-being through care.

### Theory assumptions and propositions

TECompSPRU’s assumptions include:

1. College students are a behavioral system that, through the disharmony of its subsystems, is subject to health behaviors prone to risks;
2. The college environment and the subjectivity of being a college student present stressors that favor the development of risk-prone health behaviors and/or risky behaviors;
3. Stressors in higher education can disrupt one or more subsystems directly or indirectly in a sequential manner;
4. Risk-prone health behavior precedes risky behavior, while both can predict pathologies or health problems;
5. Health practices and health behaviors can minimize the occurrence of risk-prone health behaviors and risky behaviors;
6. Risk-prone health behaviors can be prevented through nursing care;
7. Nursing care for college students is unique and differs from the care provided to other professional categories;
8. The multidimensionality of college students and the unique ways they experience risk-prone health behaviors demand care from other professional categories beyond nursing, thus enabling interprofessional collaboration.
9. Nursing care for college students, when grounded in TECompSPRU, can strengthen health behaviors, encourage healthy behaviors, and minimize the occurrence of health-prone and/or at-risk behaviors;
10. TECompSPRU is structured around eight primary and ten secondary concepts, interrelated, that describe and explain college students’ experience in the face of the stressors of higher education, the manifestation of behaviors, and the guiding principles for nursing care.

The following propositions comprise TECompSPRU:

1. Risk-prone health behavior, as well as its dimensions, is influenced by sociodemographic, economic, family, and academic variables;
2. Students attending full-time courses tend to exhibit a greater propensity for risky health behaviors due to the demands and stimuli related to the subsystems of personal, formative, and psychological development;
3. Progression through the semesters of the course is associated with an increased propensity for risk, considering the accumulation of experiences and conditions inherent to the academic training process;
4. The transition between stages of health behavior to risk-prone health behavior and risk-taking behavior is negative for college students;
5. College students who do not recognize the predictors of the phenomenon lack motivation and acceptance of a care plan;
6. A lack of motivation and poor/no acceptance of a care plan favor the development of risk-prone health behaviors and/or risky behaviors;
7. Stressors that affect risk-prone health behavior can manifest individually or collectively;
8. Maintenance factors can be enhanced through a care plan developed by the nurse, based on the Nursing Process and the use of standardized language systems;
9. The transition from stages of health conduct to health behavior is positive for college students;
10. Understanding risk-prone health behaviors and adopting healthy behaviors among college students promotes a harmonious balance between their subsystems, contributing to a reduction in risk-taking.

### Pictorial model

**Figure 3:**
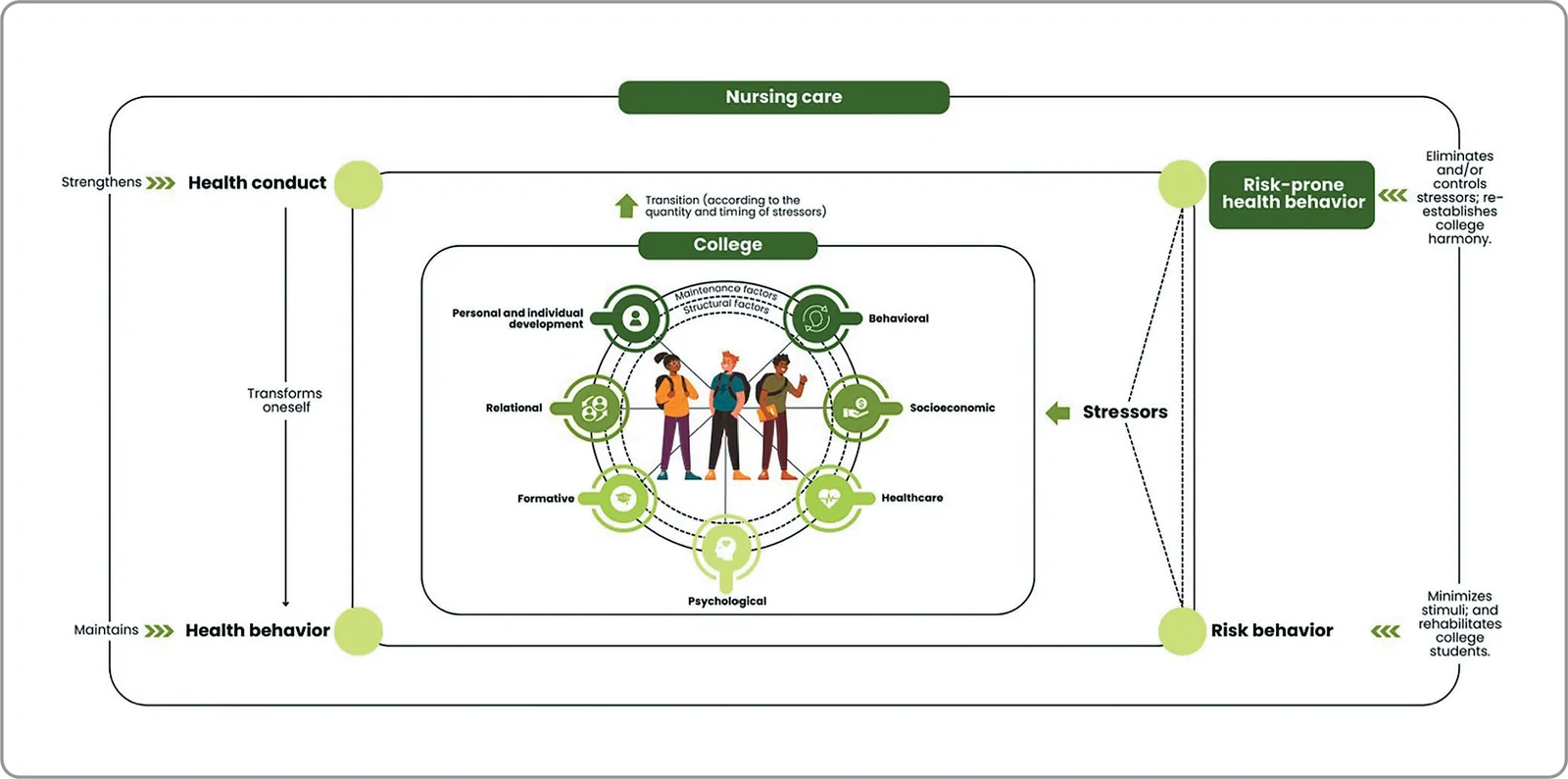
Pictogram of the Middle-Range Theory for Risk-Prone Health Behavior in College students, Iguatu, Ceará, Brazil, 2026

The relationships and purposes of TECompSPRU are presented graphically (Figure 3).

## DISCUSSION

An approach to being a college student involves engaging with the college, risk-prone health behaviors, and nursing practice in an inseparable and complementary manner.

The college constitutes a strategic space for the inclusion and strengthening of nursing care, due to the uniqueness of this phase of the life cycle, marked by transitions, vulnerabilities, and specific care demands^(12)^. Thus, a nursing theory focused on understanding this phenomenon can provide a foundation for more effective and evidence-based practices.

Entering college represents a phase of breaking with customs and intense individual transformations, whether due to the new responsibilities assumed or the heterogeneity of relationships and groups^(13)^. In this context, exposure to stressors and difficulty in coping with them can underpin a predisposition to risky behaviors. To differentiate between them, a progressive and interdependent relationship is recognized between health conduct, health behavior, risk-prone health behavior, and risky behavior. Health behavior can be understood as the state, disposition, or action resulting from personal, family, and cultural influences that guide how an individual relates to their own body and the health-disease process. Such behaviors express values, beliefs, and learning acquired throughout life, and can manifest consciously, through deliberate care decisions, or unconsciously, through habits incorporated into daily life, acting beneficially on the processes of maintaining, recovering, and promoting health^(14)^.

Health behavior, in turn, refers to a set of conscious and intentional practices aimed at maintaining, improving, or restoring health status. It involves rational choices motivated by knowledge, beliefs, and perceptions of vulnerability, and is guided by cognitive and attitudinal processes that reflect an individual’s level of self-care and health literacy^(15)^. Examples include adopting a balanced diet, engaging in physical activity, seeking preventative care, and adhering to treatments.

Risk-prone health behavior among college students represents a predisposition to an articulated set of practices that are detrimental to overall health, adopted voluntarily, unfavorably, and sometimes self-destructively. The tendency to adopt practices and habits that, even if intentionally undertaken, cause potential or actual harm to health and well-being, revealing weaknesses in self-care and decision-making skills. This includes the use of psychoactive substances, unsafe sexual practices, inadequate diet, sedentary lifestyle, sleep disturbances, among others^(16)^.

This is a complex phenomenon involving personal, relational, family, psychological, socioeconomic, environmental, and academic dimensions, demanding interdisciplinary attention, especially from nursing.

Risk-prone health behavior is considered a complex and independent human response, the analysis of which requires the conceptual delimitation of clinical indicators, causal factors, associated conditions, and at-risk population. Although it may relate to other human responses, it maintains a unique character and demands in-depth theoretical study of its constitutive dimensions and population variations. The theory proposed here contributes to understanding this construct within the context of college health, considering students as a social subject who is simultaneously a product and an agent of transformation of the environment in which they live.

Finally, risky behavior is characterized by recurring and well-defined behavioral patterns, the systematic repetition of which directly or indirectly promotes physical, mental, and social illness. Unlike risk-prone behavior, which expresses a predisposition or tendency, risky behavior represents the concretization of these actions in daily life, with the potential to generate measurable clinical, functional, or social harm^(16)^.

The literature reinforces the leading role of nursing in promoting college health, through individual and collective interventions that encourage self-care and the development of healthy environments^(17,18)^. The use of technologies, such as the Nursing Process, favors the development of skills in health promotion, reducing symptoms of stress, anxiety, and depression, and promoting academic retention and success^(19)^.

Nursing plays a central role in promoting college students’ health and in consolidating healthy universities, acting both in direct care and in the creation of institutional environments that favor physical, mental, and social well-being^(20,21)^. Nursing theories contribute to ethical and epistemological reflection on the health-disease process and, when integrated with evidence-based practice, strengthen the disciplinary field from a global perspective^(22)^.

The theoretical assumptions, understood as principles assumed to be true^(9)^, were organized into ontological, contextual, dynamic, teleological and epistemological dimensions. The ontological dimension encompasses being and its meaning^(23)^ (assumption 1); the contextual dimension covers environmental, social, and institutional conditions (assumptions 2, 4 and 5); the dynamic dimension describes the manifestation and effects of the phenomenon (assumption 3); the teleological dimension examines its purposes and reasons^(24)^ (assumptions 7, 8, 9 and 10); and the epistemological dimension grounds nursing knowledge^(23)^ (assumption 6).

In a complementary way, the propositions express hypotheses that demand empirical testing^(9)^, organized according to the following domains: contextual determinants; negative transitions; positive transitions; individual barriers to care; nursing response and equilibrium outcome; and health promotion. Contextual determinants encompass the predisposing factors of the phenomenon^(25)^ (propositions 7, 8, 9 and 10). Transition is understood as the passage from one stage to another, and can contribute to the health process (proposition 5) or favor illness (proposition 6). Individual barriers refer to factors intrinsic to individuals, which, when present, compromise the effectiveness of health processes (assumptions 2 and 3). Nursing responses relate to professional care, which articulates theory and practice and is based on ontological, epistemological and praxeological precepts^(23)^ (assumptions 4). Finally, balance and health promotion translate into the autonomous and participatory modification of behaviors, consolidating the positivity of health processes^(26)^ (assumption 1).

The application of TECompSPRU is recommended as theoretical support for the Nursing Process. Assessment can be carried out using the Questionnaire on Health Risk-Prone Behavior in University Students (*Questionário do Comportamento de Saúde Propenso a Risco em Universitários*) and the Health Risk Behavior Questionnaire for University Students (*Questionário do Comportamento de Risco em Saúde de Universitários*), both with evidence of validity for the college population. Diagnosis should be based on the NANDA-I taxonomies and/or the International Classification for Nursing Practice, guiding the planning of actions based on the Nursing Interventions Classification and the International Classification of Nursing Outcomes, as well as NANDA 360 - an expanded classification for nursing diagnoses, patient outcomes and goals, and nursing actions.

Nurses must utilize semiological, semiotechnical, and relational skills in providing care, documenting the evolution and transitions observed, in order to consolidate practices based on theory and evidence.

### Study limitations

TECompSPRU encompasses the concepts of health conduct and health behavior related to the central phenomenon, although its specificities still require theoretical and practical deepening for the refinement of its constitutive and operational elements. Stressors were identified in association with risk-prone health behaviors and risk behaviors, considering their interrelationships and the multicausal nature of the constructs, which may generate interpretative challenges for nursing professionals and reinforces the need for studies that explore causality, mediation, and confounding. Furthermore, the subsystems, built from scientific evidence and reflections on college life, may require adjustments or new conceptual units in the future, since the college context is dynamic. Therefore, continuous refinement of the concepts is recommended according to new demands and emerging evidence.

Thus, this study has inherent limitations due to its predominantly theoretical nature, since the propositions formulated have not yet been subjected to systematic empirical testing, which restricts causal and predictive inferences. The inductive stage, although strengthened by conceptual analysis and focus groups, concentrated on specific institutional contexts, which may limit the immediate generalization of the theory to different sociocultural and organizational realities of higher education. Furthermore, the derivation from the JBS, while theoretically consistent, may have favored certain conceptual frameworks over other explanatory approaches to health behavior. It should also be noted that the complexity and multidimensionality of the phenomenon may require future refinements to the secondary concepts and proposed relationships as new empirical evidence is incorporated. Thus, TECompSPRU should be understood as a theoretical proposition under consolidation, open to revisions, refinements, and expansions resulting from its application in research and clinical practice.

### Contributions to nursing, health, or public policy

TECompSPRU contributes to understanding risk-prone health behavior as a phenomenon embedded in the college context that demands systematized nursing care, by offering a robust conceptual framework, supported by multiple research methods, capable of guiding clinical reasoning, judgment, and intervention planning. By overcoming reductionist perspectives, it enables an integrated analysis that considers individuals, their components, the stimulating factors, and the different stages of behavior, providing direction to nursing practice and strengthening the articulation between theory and practice. Its construct has the potential to impact the epistemological and ontological field of nursing in college health by fostering reflections on individuality, context, and the relationships that precede behaviors, as well as promoting better health outcomes, such as the reduction of chronic non-communicable diseases and the adoption of healthy behaviors among college students.

## FINAL CONSIDERATIONS

TECompSPRU is composed of eight primary concepts, ten secondary concepts, ten assumptions, and ten propositions that, in an integrated way, seek to explain and describe college students, their constitutive dimensions, and the manifestation of health conduct, health behaviors, risk-prone health behaviors, and risk behaviors. This theory does not exhaust the study of the phenomenon. Therefore, its adoption is proposed as an epistemological provocation for the improvement of knowledge in nursing and the construction of counterpoints, reaffirming the dynamic nature of knowledge and care as practices in continuous development and reconstruction.

Validating TECompSPRU requires subsequent empirical and applied steps capable of testing the internal consistency, practical utility, and explanatory power of the formulated propositions. Initially, empirical testing of the propositions is recommended through analytical observational studies (cross-sectional and longitudinal) that allow for verifying associations between stressors, subsystems, behavioral transitions, and health outcomes. Subsequently, statistical and structural modeling studies can be employed to assess the plausibility of the proposed relationships and the directionality of the effects between the central concepts. Another relevant strategy consists of the clinical and pragmatic validity of the theory, through its application in the Nursing Process in college healthcare services, analyzing its ability to guide diagnoses, interventions, and outcomes sensitive to nursing practice. Furthermore, cross-cultural and inter-institutional validity studies, conducted in different college contexts, could enhance the external robustness of the theory. Finally, it is recommended that theoretical assessments be carried out by experts, aiming at a critical analysis of the conceptual clarity, parsimony, logical consistency, and epistemological adequacy of TECompSPRU as an MRT.

## Data Availability

The research data are not available.
