# Supplementary material for: Middle-Range Theory for Risk-Prone Health Behavior in College Students
Source: Rev Bras Enferm. 2026 Sep 21;79:e20250664. doi: 10.1590/0034-7167-2025-0664 (PMC13592784; doi:10.1590/0034-7167-2025-0664)
Supplement: Translation

# Translation: Teoria de Médio Alcance para o Comportamento de Saúde Propenso a Riscos em Universitários

**DOI:** 10.1590/0034-7167-2025-0664pt

**Authors:** Pedro Lucas Ferreira Mota, Lucas Dias Soares Machado, Adriana de Moraes Bezerra, Maria Sinara Farias, Viviane Martins da Silva, Nayara Santana Brito

**Affiliations:** I Universidade Federal do Ceará. Fortaleza, Ceará, Brasil; II Universidade Regional do Cariri. Iguatu, Ceará, Brasil; III Universidade Regional do Cariri. Crato, Ceará, Brasil; IV Universidade Estadual Vale do Acaraú. Sobral, Ceará, Brasil

**Keywords:** Teoria de Enfermagem, Comportamentos de Risco à, Saúde, Estudantes, Universidades, Enfermagem.

## Abstract

**Objetivos:**

descrever o desenvolvimento de uma Teoria de Médio Alcance para o Comportamento de Saúde Propenso a Riscos em Universitários.

**Métodos:**

estudo teórico orientado para a elaboração de uma teoria a partir da combinação de técnicas de indução por meio de análise conceitual e grupo focal, e da derivação de conceitos da Teoria do Sistema Comportamental de Dorothy Johnson.

**Resultados:**

a teoria articula oito conceitos primários, dez conceitos secundários, dez pressupostos e dez proposições que, em conjunto, buscam descrever e explicar o contexto universitário, suas dimensões constitutivas e a manifestação de condutas de saúde, comportamentos de saúde, comportamentos de saúde propensos a risco e comportamentos de risco.

**Considerações Finais:**

a teoria fornece uma base teórico-prática que supera a visão reducionista dos comportamentos de saúde propensos a riscos em universitários, promovendo uma análise integrada do indivíduo, de seus determinantes e estágios comportamentais e orientando a prática da enfermagem.

## INTRODUÇÃO

O processo de adaptação à universidade é complexo e exige do acadêmico diversos ajustes na rotina, além da adoção e manutenção de hábitos saudáveis e do enfrentamento de obstáculos, sobretudo no campo da saúde^(1)^. Reconhece-se como produto desse processo a manifestação de comportamentos de risco à saúde, caracterizados pela predisposição a morbidades decorrentes de práticas comuns nesse público, como sedentarismo^(2)^, hábitos alimentares inadequados^(3)^, padrão de sono prejudicado^(4)^, condutas sexuais de risco, abuso de substâncias e comportamentos perigosos no trânsito^(5)^. Apesar da ampla produção sobre esses comportamentos, ainda são escassos os modelos teóricos que expliquem, de forma sistematizada, os mecanismos que predispõem o universitário a práticas de risco, especialmente sob a ótica da enfermagem.

A compreensão, o reconhecimento e a atuação do enfermeiro em relação aos comportamentos de saúde de risco entre universitários demonstram sensibilidade às necessidades desse grupo e o estabelecimento de estratégias antecipadas que favoreçam o bem-estar de acordo com suas demandas reais. Torna-se, portanto, essencial a implementação de ações assertivas e transformadoras, capazes de sustentar práticas de cuidado coerentes com a singularidade desse público^(6)^.

Para a efetivação das ações, propõe-se a utilização do Processo de Enfermagem como ferramenta metodológica, organizativa e dinâmica, estruturada em cinco etapas inter-relacionadas, interdependentes, recorrentes e cíclicas, quais sejam: avaliação; diagnóstico de enfermagem; planejamento; implementação; e evolução de enfermagem^(7)^. Por sua vez, faz-se relevante que esse processo esteja ancorado em teorias de enfermagem que descrevam, expliquem, prevejam ou prescrevam as condições e conexões de um fenômeno, como os comportamentos de saúde propensos a riscos em universitários, qualificando a prática clínica e fortalecendo a identidade disciplinar^(8)^.

Embora se observem avanços significativos no campo teórico da enfermagem, a aplicação desses referenciais ao cuidado do universitário permanece incipiente. Essa lacuna evidencia a necessidade de construir um modelo específico que subsidie a atuação do enfermeiro e amplie a compreensão do processo de saúde-doença nesse contexto.

As Teorias de Médio Alcance (TMAs) se destacam nesse cenário pela aplicabilidade prática e pelo potencial de aproximar teoria e prática. Conceitualmente, abrangem um conjunto de conceitos com menor nível de abstração e definições operacionais, constituindo proposições concretas e passíveis de verificação empírica^(9,10)^. Essa característica reforça seu valor epistemológico para o avanço do conhecimento em enfermagem.

Até o momento, não há registros de TMAs voltadas especificamente ao comportamento de saúde propenso a riscos em universitários que integrem conceitos e definições destinados à descrição e à explicação desse fenômeno e de suas relações com os múltiplos estágios do processo de saúde-doença. Essa lacuna fundamenta a proposta deste estudo, evidenciando seu caráter inédito e inovador.

Postula-se, portanto, que o desenvolvimento de uma TMA voltada ao comportamento de saúde propenso a riscos em universitários permitirá construir um arcabouço teórico capaz de descrever e explicar as unidades que compõem o fenômeno, bem como suas inter-relações com outras categorias do comportamento humano. Além disso, essa teoria apresentará potencial para fundamentar as ações de enfermagem e intervir nos fatores que predizem e ocasionam o adoecimento.

A abordagem proposta também se articula aos Objetivos de Desenvolvimento Sustentável (ODS), especialmente ao ODS 3 (Saúde e Bem-Estar), que visa assegurar uma vida saudável e promover o bem-estar para todas as pessoas, em todas as idades. Mais especificamente, ela se relaciona ao ODS 3.5, que propõe o fortalecimento da prevenção e do tratamento do abuso de substâncias, e ao ODS 3.8, que busca garantir cobertura universal de saúde, bem como acesso a serviços e medicamentos de qualidade a preços acessíveis. Essas conexões reforçam a pertinência social e política da proposta ao tratar do comportamento de saúde propenso a riscos e de suas implicações para o cuidado em saúde.

## OBJETIVOS

Descrever o desenvolvimento de uma TMA para o comportamento de saúde propenso a riscos em universitários.

## MÉTODOS

### Aspectos éticos

A pesquisa foi aprovada pelo Comitê de Ética em Pesquisa da Universidade Regional do Cariri segundo Certificado de Apresentação para Apreciação Ética n^o^ 78484824.3.0000.5055.

### Referencial teórico

O presente estudo adota o Sistema Comportamental de Dorothy Johnson (em inglês, *Johnson Behavioral System* - JBS) como referencial teórico, visto que tal sistema articula conceitos como subsistemas, componentes estruturais e requisitos funcionais, que permitem explicitar as relações do indivíduo, enquanto sistema comportamental, consigo mesmo, entre seus subsistemas e com o ambiente. Essa teoria evidencia a enfermagem como força promotora de equilíbrio, capaz de intervir em disfunções comportamentais e favorecer a sua harmonia^(11)^, configurando-se como um alicerce pertinente para o fenômeno em estudo. Seus escritos se inserem na era dos sistemas, marcada pela busca de entendimento do todo, e visavam reafirmar a enfermagem como uma profissão autônoma e distinta das demais áreas da saúde.

### Tipo de estudo

Trata-se de um estudo teórico, orientado para elaboração de uma TMA, a partir da combinação de técnicas de indução e derivação.

### Procedimentos metodológicos

O processo de teorização articulou técnicas de indução por meio de análise conceitual e grupos focais, além de derivação de uma grande teoria^(10)^.

A análise conceitual buscou concepções na literatura científica sobre o comportamento de saúde propenso a riscos em universitários via revisão integrativa, resultando na delimitação das unidades que compõem o conceito^(12)^. Nesse sentido, os antecedentes, atributos, consequentes, definições conceituais, casos e indicadores empíricos foram incorporados à teorização.

Na perspectiva de compreender a subjetividade da manifestação do fenômeno, realizaram-se grupos focais com estudantes de uma universidade pública do estado do Ceará e profissionais de cuidado ao universitário de uma instituição pública da Paraíba. A utilização de participantes de instituições distintas ocorreu pelo vínculo dos autores durante o desenvolvimento do estudo, o acesso aos universitários em uma das instituições e a existência dos núcleos de cuidado estudantil na outra. Assim, registrou-se a participação de 30 universitários em três grupos, com tempo médio de 46 minutos, e 16 profissionais representantes dos núcleos de cuidado estudantil dos *campi* da instituição de ensino, em grupo único, com duração aproximada de duas horas. A saturação de dados e a temática demarcaram o fim da coleta com os universitários, enquanto a representação de 11 *campi* da instituição da Paraíba foi alcançada no momento com os profissionais (52,3%).

Os *corpora* textuais foram analisados no *Interface de R pour les Analyses Multidimensionnelles de Textes et de Questionnaires* segundo a Classificação Hierárquica Descendente. Dessa forma, as concepções do fenômeno foram consideradas na teorização para aperfeiçoamento das definições, bem como nas relações entre os comportamentos.

Assim, o material advindo da análise de conceito e grupos focais sustentou a formulação das afirmações, que têm o papel de alicerçar a concepção da teoria. Essas afirmações podem ser relacionais, estabelecendo conexões entre dois ou mais conceitos de uma teoria e revelando correlações, ou não relacionais, constituindo-se como definições teóricas ou operacionais de um conceito^(10)^.

Por fim, realizaram-se aproximações entre o arcabouço primário e o JBS, complementando-os, contrapondo-os ou reforçando-os, com base no aparato teórico e empírico. Esse processo caracteriza-se como derivação, uma estratégia criativa baseada em analogia e na transposição de ideias de um campo teórico para a explicação e/ou predição em um novo campo^(10)^.

Na etapa final, realizou-se a modelagem teórica, resultando em um esquema pictórico da TMA. Como produto, apresentaram-se a teoria, suas finalidades, conceitos e afirmações não relacionais, afirmações relacionais entre os conceitos, pressupostos, proposições e o modelo pictorial, possibilitando a compreensão da contextualização da teoria e de suas possibilidades de aplicação.

## RESULTADOS

### Teoria de Médio Alcance para o Comportamento de Saúde Propenso a Riscos em Universitários

A Teoria de Médio Alcance para o Comportamento de Saúde Propenso a Riscos em Universitários (TECompSPRU) é uma teoria científica de nível de abstração intermediário (TMA), de caráter descritivo e explicativo, desenvolvida sob a perspectiva *etic* (externa), que incorporou evidências de estudos com universitários realizados em diferentes contextos nacionais e internacionais, e a perspectiva *emic* (interna), que envolveu as subjetividades de profissionais que atuam no cuidado ao universitário e de representantes da própria população de interesse. Essas perspectivas subsidiaram o propósito de contribuir para a formulação, a consolidação e a ampliação do corpo de conhecimentos da enfermagem contemporânea, com foco no cuidado ao estudante universitário, precedendo o desenvolvimento teórico.

### Finalidades da teoria

A TECompSPRU busca descrever e explicar as relações entre o universitário e a manifestação de comportamentos, especialmente do tipo comportamento de saúde propenso a riscos, visando fornecer um escopo de fundamentos para a compreensão do construto e do cuidado de enfermagem.

### Conceitos e afirmações não relacionais

A identificação dos conceitos precede a formulação das definições. Nesse sentido, com base nas evidências construídas ao longo do estudo, delimitaram-se conceitos primários (universitário; subsistemas; estressores; conduta de saúde; comportamento de saúde; comportamento de saúde propenso a riscos; comportamento de risco; e cuidado de enfermagem) e secundários (subsistema do desenvolvimento pessoal e individual; subsistema relacional; subsistema formativo; subsistema psicológico; subsistema comportamental; subsistema socioeconômico; subsistema do cuidado em saúde; fatores estruturais; fatores de manutenção; e transição). Formularam-se as definições conceituais, especialmente adaptando as unidades centrais dos conceitos sistema comportamental (representado nesta teoria pelo conceito universitário), subsistemas e estressores, abordados por Dorothy Johnson em sua teoria.

Portanto, listaram-se a seguir as definições conceituais que integram a TECompSPRU.

Alinhados entre si, o sistema se refere a uma totalidade que se estabiliza pela adequação de suas partes, e o comportamento constitui respostas voluntárias ou involuntárias adjuradas pelo meio, quer seja o ser pessoal ou meio de interação. Ao passo, o universitário se refere a um sistema comportamental que, pela interação de suas partes, produz respostas voluntárias ou involuntárias, repetitivas e/ou organizadas, capazes de estabelecer relações com a coletividade e o meio.

Os subsistemas são unidades conectadas, balizadas na individualidade e nos eventos do ensino superior, responsáveis pela harmonia do todo. Esses são pontuados e definidos conforme Figura 1.

**Figura 1:** Definições dos subsistemas que compõem a Teoria de Médio Alcance para o Comportamento de Saúde Propenso a Riscos em Universitários, Iguatu, Ceará, Brasil, 2026

O funcionamento dos subsistemas é mediado pelos fatores estruturais, envolvendo a criticidade e a avaliação entre: situação (estímulo/estressor); julgamento (avaliação da situação e estabelecimento de metas); e comportamento (operacionalização). De modo complementar, os fatores de manutenção ofertam sustentação para manutenção da eficácia comportamental, compreendendo três elementos: (1) conhecimento em saúde (favorecem condutas de saúde e comportamentos de saúde); (2) recursos sociais (possibilitam a adoção de condutas de saúde); e (3) apoio (envolve o incentivo ambiental/institucional e das relações).

Por sua vez, os estressores são estímulos desfavoráveis provenientes do ser ou do ambiente universitário, que se associam ao comportamento de saúde propenso a riscos e/ou ao comportamento de risco, dada a multicausalidade e a indissociabilidade de seus constituintes. Ou seja, as unidades que compõe os três eixos, constantemente, desencadearão novos comportamentos.

Quanto aos possíveis estágios em que o universitário pode transitar a partir de suas atitudes, destacam-se: a conduta de saúde, que é o estado ou a ação advinda da formação pessoal, familiar e/ou cultural que, conscientemente ou não, atua beneficamente nos processos de saúde; o comportamento de saúde, que se refere a um conjunto de práticas conscientes que mantêm ou favorecem o cuidado com a saúde; o comportamento de saúde propenso a riscos, delineado como tendências à adoção de hábitos prejudiciais à saúde e ao bem-estar, de caráter voluntário, negativo, desfavorável e/ou destrutivo; e o comportamento de risco, caracterizado como padrões comportamentais recorrentes e delimitados que promovem o adoecimento.

Transição é a capacidade em que o universitário percorre entre os estágios de conduta de saúde, comportamento de saúde, comportamento de saúde propenso a riscos e comportamento de risco. Esse percurso pode ser mediado por: (1) quantidade de estímulos - único (envolve um estímulo) ou múltiplo (envolve mais de um estímulo); e (2) temporalidade - pontual (acontece em um período curto, como horas e dias), limitado (período bem definido dentro da programação vital e organização do sistema) e constante (período de duração maior, por vezes recorrentes e com prazos pouco delimitados).

Por conseguinte, o cuidado de enfermagem envolve condutas ordenadas e sistemáticas, baseadas no raciocínio clínico e na tomada de decisão, voltadas para a prevenção (minimização dos estímulos de adoecimento), promoção (aprimoramento da qualidade de vida e autonomia para enfrentamento dos preditores que favorecem o adoecimento) e recuperação (auxílio e manejo diante do adoecimento) da saúde do universitário. Devem estar mediadas pelas ferramentas organizativas e de gestão da prática de enfermagem, sendo implementadas principalmente na manutenção ou no restabelecimento da harmonia do universitário.

Atrelado a esses conceitos, estabeleceu-se conexão com o metaparadigma da enfermagem de Johnson (pessoa, saúde, ambiente e enfermagem), haja vista sua essencialidade na conformação dos limites do conhecimento da disciplina em comparação com outros saberes da saúde. (Quadro 1).

**Quadro 1:** Representações do metaparadigma da enfermagem de Johnson na Teoria de Médio Alcance para o Comportamento de Saúde Propenso a Riscos em Universitários, Iguatu, Ceará, Brasil, 2026

Metaparadigma da enfermagem	Definição na Teoria de Médio Alcance para o Comportamento de Saúde Propenso a Riscos em Universitários
Pessoa	O universitário é um sistema comportamental subjetivo, adaptável, singular e relacional, cuja interação com a universidade predispõe à ocorrência de comportamentos de saúde propensos a riscos.
Saúde	Integra o estado particular e a busca pelo bem-estar e harmonia do universitário em seus múltiplos aspectos, exigindo que o cuidado esteja articulado com a prevenção e a promoção da saúde diante dos fatores que predizem o fenômeno ou a recuperação da saúde em situações de adoecimento.
Ambiente	Considera a individualidade do universitário para a manifestação de respostas humanas, bem como os estímulos moderadores ou confundidores do ensino superior confluentes para a ocorrência do evento.
Enfermagem	É a ciência do cuidado holístico, baseada em fundamentos teórico-práticos e distinta da medicina. Aborda integralmente o universitário em suas dimensões física, emocional e social. Seu escopo abrange o acolhimento, o mapeamento de preditores, o raciocínio clínico, a tomada de decisão e a aplicação de intervenções alinhadas à redução/controle dos fatores que potencializam o evento, bem como à recuperação de condições de adoecimento associadas.

### Afirmações relacionais entre os conceitos da teoria

As relações conceituais foram, em síntese, apresentadas na Figura 2.

**Figura 2:** Afirmações relacionais entre os conceitos que compõem a Teoria de Médio Alcance para o Comportamento de Saúde Propenso a Riscos em Universitários, Iguatu, Ceará, Brasil, 2026

O universitário é um sistema comportamental que, por intermédio da harmonia (saúde) ou desarmonia (doença/agravo) entre os seus subsistemas, quer seja por estímulos do ser universitário ou do meio universitário (ambiente), assume atitudes alinhadas às condutas de saúde, ao comportamento em saúde, ao comportamento de saúde propenso a riscos e/ou ao comportamento de risco.

O comportamento de saúde propenso a riscos é tido como o eixo de atenção nessa proposição, dado que a minimização da sua ocorrência transporta o universitário (pessoa) aos comportamentos que favorecem a saúde, ou seja, reduz o comportamento de risco e, consequentemente, o adoecimento. Ao passo, a exacerbação desses comportamentos favorece o adoecimento.

Dessa maneira, o enfermeiro enquanto profissional holístico e competente à implementação do cuidado de enfermagem (enfermagem) deve proporcionar ao universitário estratégias que eliminem os estressores, promova saúde (manutenção da saúde) e, em casos de recorrência dos comportamentos de propensão ao risco, atuem de modo a fornecer subsídios à recuperação da saúde. É relevante que o cuidado de enfermagem esteja alinhado às atribuições éticas da categoria e fundamentado em evidências científicas, devendo envolver desde o acolhimento e a identificação dos estressores até a prescrição e averiguação da efetividade do plano de cuidado. Assim, o objetivo da enfermagem é compreender os fatores que desestabilizam o universitário e favorecer o processo de saúde e bem-estar por meio do cuidado.

### Pressupostos e proposições da teoria

Os pressupostos da TECompSPRU incluem:

1. O universitário é um sistema comportamental que, por intermédio da desarmonia de seus subsistemas, está sujeito ao comportamento de saúde propenso a riscos;
2. O ambiente universitário e a subjetividade do ser universitário apresentam estressores que favorecem o desenvolvimento do comportamento de saúde propenso a riscos e/ou comportamento de risco;
3. Os estressores do ensino superior podem desarmonizar um ou mais subsistemas direta ou indiretamente de modo sequencial;
4. O comportamento de saúde propenso a riscos precede o comportamento de risco, ao passo que ambos podem predizer patologias ou agravos à saúde;
5. As condutas de saúde e o comportamento em saúde podem minimizar a ocorrência do comportamento de saúde propenso a riscos e do comportamento de risco;
6. O comportamento de saúde propenso a riscos pode ser evitado por meio do cuidado de enfermagem;
7. O cuidado de enfermagem ao universitário é único e difere do cuidado das demais categorias profissionais;
8. A multidimensionalidade do universitário e as singularidades em vivenciar o comportamento de saúde propenso a riscos demandam cuidados de outras categorias profissionais para além da enfermagem, efetuando a interprofissionalidade;
9. O cuidado de enfermagem ao universitário, quando fundamentado na TECompSPRU, pode fortalecer as condutas de saúde, estimular o comportamento em saúde e minimizar a ocorrência do comportamento de saúde propenso e/ou risco;
10. A TECompSPRU é estruturada em oito conceitos primários e dez conceitos secundários, inter-relacionados, que descrevem e explicam a experiência do universitário diante dos estressores do ensino superior, a manifestação de comportamentos e os eixos orientadores para o cuidado de enfermagem.

Compõem a TECompSPRU as seguintes proposições:

1. O comportamento de saúde propenso a riscos, assim como suas dimensões, é influenciado por variáveis sociodemográficas, econômicas, familiares e acadêmicas;
2. Estudantes que frequentam cursos em turno integral tendem a manifestar maior propensão ao comportamento de saúde de risco em virtude das demandas e estímulos relacionados aos subsistemas de desenvolvimento pessoal, formativo e psicológico;
3. A progressão nos semestres do curso se associa ao aumento da propensão ao risco, considerando-se o acúmulo de experiências e condições inerentes ao processo de formação acadêmica;
4. A transição entre os estágios de comportamento de saúde para o comportamento de saúde propenso a riscos e comportamento de risco é negativa ao universitário;
5. O universitário que não reconhece os preditores do fenômeno não apresenta motivação e aceitabilidade do plano de cuidado;
6. O déficit de motivação e pouca/nenhuma aceitação do plano de cuidados favorecem o desenvolvimento de comportamento de saúde propenso a riscos e/ou comportamento de risco;
7. Os estressores ao comportamento de saúde propenso a riscos podem se apresentar de forma individualizada ou coletiva;
8. Os fatores de manutenção podem ser potencializados por meio do plano de cuidado elaborado pelo enfermeiro, fundamentado no Processo de Enfermagem e no uso de sistemas de linguagem padronizada;
9. A transição entre os estágios de conduta de saúde para o comportamento de saúde é positiva ao universitário;
10. A compreensão do comportamento de saúde propenso a riscos e a adoção de comportamentos de saúde pelo universitário favorecem o equilíbrio harmônico entre os seus subsistemas, contribuindo para a redução da propensão a riscos.

### Modelo pictorial

Apresentam-se graficamente as relações e propósitos da TECompSPRU (Figura 3).

**Figura 3:** Pictograma da Teoria de Médio Alcance para o Comportamento de Saúde Propenso a riscos em Universitários, Iguatu, Ceará, Brasil, 2026

## DISCUSSÃO

A abordagem ao ser universitário implica aproximações com a universidade, comportamentos de saúde propensos a riscos e com a prática de enfermagem, de modo indissociável e complementar.

A universidade constitui um espaço estratégico para a inserção e o fortalecimento do cuidado de enfermagem, devido à singularidade dessa fase do ciclo vital, marcada por transições, vulnerabilidades e demandas específicas de cuidado^(12)^. Assim, uma teoria de enfermagem voltada à compreensão desse fenômeno pode fundamentar práticas mais efetivas e baseadas em evidências.

O ingresso na universidade representa uma fase de ruptura de costumes e de intensas transformações individuais, seja pelas novas responsabilidades assumidas, seja pela heterogeneidade das relações e grupos^(13)^. Nesse contexto, a exposição a estressores e a dificuldade em enfrentá-los podem sustentar a predisposição a comportamentos de risco. Para diferenciá-los, reconhece-se uma relação progressiva e interdependente entre conduta de saúde, comportamento de saúde, comportamento de saúde propenso a riscos e comportamento de risco. A conduta de saúde pode ser compreendida como o estado, a disposição ou a ação resultante de influências pessoais, familiares e culturais, que orientam o modo como o indivíduo se relaciona com o próprio corpo e com o processo de saúde-doença. Tais condutas expressam valores, crenças e aprendizagens adquiridas ao longo da vida, e podem se manifestar de forma consciente, por meio de decisões deliberadas de cuidado, ou inconsciente, mediante hábitos incorporados ao cotidiano, atuando beneficamente sobre os processos de manutenção, recuperação e promoção da saúde^(14)^.

O comportamento de saúde, por sua vez, refere-se a um conjunto de práticas conscientes e intencionais que visam conservar, melhorar ou restabelecer o estado de saúde. Envolve escolhas racionais e motivadas por conhecimentos, crenças e percepções de vulnerabilidade, sendo orientado por processos cognitivos e atitudinais que refletem o nível de autocuidado e o letramento em saúde do indivíduo^(15)^. Exemplos incluem a adoção de alimentação equilibrada, a prática de atividades físicas, a busca por atendimento preventivo e a adesão a tratamentos.

O comportamento de saúde propenso a riscos em universitários representa a predisposição a um conjunto articulado de práticas prejudiciais à saúde em sua integralidade, assumidas de modo voluntário, desfavorável e, por vezes, autodestrutivo. A tendência à adoção de práticas e hábitos que, ainda que intencionalmente assumidos, acarretam prejuízos potenciais ou efetivos à saúde e ao bem-estar, revelando fragilidades nas competências de autocuidado e tomada de decisão. Incluem-se nesse escopo o uso de substâncias psicoativas, práticas sexuais inseguras, alimentação inadequada, sedentarismo, desregulação do sono, entre outros^(16)^.

Trata-se de um fenômeno complexo que envolve dimensões pessoais, relacionais, familiares, psicológicas, socioeconômicas, ambientais e acadêmicas, demandando atenção interdisciplinar, especialmente da enfermagem.

Considera-se o comportamento de saúde propenso a riscos como uma resposta humana complexa e independente, cuja análise requer a delimitação conceitual de indicadores clínicos, fatores causais, condições associadas e população em risco. Embora possa se relacionar a outras respostas humanas, mantém caráter singular e demanda aprofundamento teórico sobre suas dimensões constitutivas e variações populacionais. A teoria aqui proposta contribui para compreender esse construto no âmbito da saúde universitária, considerando o estudante como sujeito social que é, simultaneamente, produto e agente de transformação do meio em que vive.

Por fim, o comportamento de risco é caracterizado por padrões comportamentais recorrentes e delimitados, cuja repetição sistemática promove direta ou indiretamente o adoecimento físico, mental e social. Diferentemente do comportamento propenso a riscos, que expressa uma predisposição ou tendência, o comportamento de risco representa a concretização dessas ações no cotidiano, com potencial de gerar danos clínicos, funcionais ou sociais mensuráveis^(16)^.

A literatura reforça o protagonismo da enfermagem na promoção da saúde universitária, mediante intervenções individuais e coletivas que estimulam o autocuidado e o desenvolvimento de ambientes saudáveis^(17,18)^. O uso de tecnologias, como o Processo de Enfermagem, favorece o desenvolvimento de competências em promoção da saúde, reduzindo sintomas de estresse, ansiedade e depressão, e promovendo a permanência e o sucesso acadêmico^(19)^.

A enfermagem exerce papel central na promoção da saúde de universitários e na consolidação de universidades saudáveis, atuando tanto no cuidado direto quanto na criação de ambientes institucionais que favorecem o bem-estar físico, mental e social^(20,21)^. As teorias de enfermagem auxiliam na reflexão ética e epistemológica sobre o processo de saúde-doença e, quando integradas à prática baseada em evidências, fortalecem o campo disciplinar em perspectiva global^(22)^.

Os pressupostos teóricos, entendidos como princípios assumidos como verdadeiros^(9)^, foram organizados nas dimensões ontológica, contextual, dinâmica, teleológica e epistemológica. A dimensão ontológica compreende o ser e seu sentido^(23)^ (pressuposto 1); a dimensão contextual abrange as condições ambientais, sociais e institucionais (pressupostos 2, 4 e 5); a dimensão dinâmica descreve a manifestação e os efeitos do fenômeno (pressuposto 3); a dimensão teleológica examina suas finalidades e razões^(24)^ (pressupostos 7, 8, 9 e 10); e a dimensão epistemológica fundamenta o conhecimento da enfermagem^(23)^ (pressuposto 6).

De modo complementar, as proposições expressam hipóteses que demandam testagem empírica^(9)^, organizadas segundo os seguintes domínios: determinantes contextuais; transições negativas; transições positivas; barreiras individuais ao cuidado; resposta de enfermagem e resultado de equilíbrio; e promoção da saúde. Os determinantes contextuais abrangem os fatores predisponentes do fenômeno^(25)^ (proposições 7, 8, 9 e 10). A transição é compreendida como a passagem de um estágio a outro, podendo contribuir para o processo de saúde (proposição 5) ou favorecer o adoecimento (proposição 6). As barreiras individuais se referem aos fatores intrínsecos ao ser, que, quando presentes, comprometem a efetividade dos processos de saúde (pressupostos 2 e 3). As respostas de enfermagem dizem respeito ao cuidado profissional, que articula teoria e prática e se alicerça em preceitos ontológicos, epistemológicos e praxiológicos^(23)^ (pressupostos 4). Por fim, o equilíbrio e a promoção da saúde traduzem a modificação autônoma e participativa dos comportamentos, consolidando a positividade dos processos de saúde^(26)^ (pressuposto 1).

Recomenda-se a aplicação da TECompSPRU como suporte teórico ao Processo de Enfermagem. A avaliação pode ser realizada com os instrumentos Questionário do Comportamento de Saúde Propenso a Risco em Universitários e Questionário do Comportamento de Risco em Saúde de Universitários, ambos com evidências de validade para o público universitário. O diagnóstico deve se apoiar nas taxonomias da NANDA-I e/ou na Classificação Internacional para a Prática de Enfermagem, orientando o planejamento das ações com base na Classificação das Intervenções de Enfermagem e na Classificação Internacional dos Resultados de Enfermagem, bem como na NANDA 360 - uma classificação ampliada para diagnósticos de enfermagem, resultados e metas do paciente, e ações de enfermagem.

O enfermeiro deve mobilizar competências semiológicas, semiotécnicas e relacionais na execução do cuidado, documentando a evolução e as transições observadas, de modo a consolidar práticas baseadas em teoria e evidências.

### Limitações do estudo

A TECompSPRU abrange os conceitos de conduta de saúde e comportamento em saúde relacionados ao fenômeno central, embora suas especificidades ainda demandem aprofundamento teórico e prático para o refinamento de seus elementos constitutivos e operacionais. Os estressores foram identificados em associação aos comportamentos de saúde propensos a riscos e aos comportamentos de risco, considerando suas inter-relações e a natureza multicausal dos construtos, o que pode gerar desafios interpretativos para o profissional de enfermagem e reforça a necessidade de estudos que explorem causalidade, mediação e confundimento. Ademais, os subsistemas, construídos a partir de evidências científicas e reflexões sobre a vivência universitária, poderão futuramente requerer ajustes ou novas unidades conceituais, uma vez que o contexto universitário é dinâmico. Assim, recomenda-se o contínuo refinamento dos conceitos conforme novas demandas e evidências emergentes.

Assim, este estudo apresenta limites inerentes à sua natureza eminentemente teórica, uma vez que as proposições formuladas ainda não foram submetidas à testagem empírica sistemática, o que restringe inferências causais e preditivas. A etapa indutiva, embora fortalecida pela análise conceitual e por grupos focais, concentrou-se em contextos institucionais específicos, o que pode limitar a generalização imediata da teoria para diferentes realidades socioculturais e organizacionais do ensino superior. Além disso, a derivação a partir do JBS, embora teoricamente consistente, pode ter privilegiado determinadas estruturas conceituais em detrimento de outras abordagens explicativas do comportamento em saúde. Ressalta-se, ainda, que a complexidade e a multidimensionalidade do fenômeno podem demandar refinamentos futuros nos conceitos secundários e nas relações propostas, à medida que novas evidências empíricas sejam incorporadas. Assim, a TECompSPRU deve ser compreendida como uma proposição teórica em consolidação, aberta a revisões, refinamentos e ampliações decorrentes de sua aplicação em pesquisa e prática clínica.

### Contribuições para a área da enfermagem, saúde ou políticas públicas

A TECompSPRU contribui para compreender o comportamento de saúde propenso a riscos como um fenômeno inserido no contexto universitário que demanda cuidado sistematizado de enfermagem, ao oferecer um arcabouço conceitual robusto, sustentado por múltiplos métodos de investigação, capaz de orientar o raciocínio clínico, o julgamento e o planejamento das intervenções. Ao superar perspectivas reducionistas, possibilita uma análise integrada que considera o indivíduo, seus componentes, os fatores estimuladores e os diferentes estágios do comportamento, conferindo direção ao fazer de enfermagem e fortalecendo a articulação entre teoria e prática. Seu construto apresenta potencial para impactar o campo epistemológico e ontológico da enfermagem na saúde universitária ao fomentar reflexões sobre a individualidade, o contexto e as relações que antecedem os comportamentos, além de favorecer melhores resultados em saúde, como a redução de doenças crônicas não transmissíveis e a adoção de comportamentos saudáveis entre universitários.

## CONSIDERAÇÕES FINAIS

A TECompSPRU é composta por oito conceitos primários, dez conceitos secundários, dez pressupostos e dez proposições que, de forma integrada, buscam explicar e descrever o universitário, suas dimensões constitutivas e a manifestação de condutas de saúde, comportamentos de saúde, comportamentos de saúde propenso a riscos e comportamentos de risco. Essa teoria não esgota o estudo do fenômeno; portanto, propõe-se sua adoção como provocação epistemológica para o aprimoramento do conhecimento em enfermagem e a construção de contrapontos, reafirmando a natureza dinâmica do saber e do cuidado como práticas em contínuo desenvolvimento e reconstrução.

A validação da TECompSPRU requer etapas subsequentes de natureza empírica e aplicada, capazes de testar a consistência interna, a utilidade prática e o poder explicativo das proposições formuladas. Inicialmente, recomenda-se a testagem empírica das proposições, por meio de estudos observacionais analíticos (transversais e longitudinais) que permitam verificar associações entre estressores, subsistemas, transições comportamentais e desfechos em saúde. Em seguida, estudos de modelagem estatística e estrutural podem ser empregados para avaliar a plausibilidade das relações propostas e a direcionalidade dos efeitos entre os conceitos centrais. Outra estratégia relevante consiste na validação clínica e pragmática da teoria, por meio de sua aplicação no Processo de Enfermagem em serviços de atenção à saúde do universitário, analisando-se sua capacidade de orientar diagnósticos, intervenções e resultados sensíveis à prática de enfermagem. Ademais, estudos de validação transcultural e interinstitucional, conduzidos em diferentes contextos universitários, poderão ampliar a robustez externa da teoria. Por fim, recomenda-se a realização de avaliações teóricas por especialistas, visando à análise crítica da clareza conceitual, parcimônia, consistência lógica e adequação epistemológica da TECompSPRU enquanto TMA.

## Data Availability

Os dados de pesquisa não estão disponíveis.
